# Preliminary Experimental Study on the Cross-Specimen Borehole Applicability of Identical Rock-Mass Characteristics Using Built-In Multi-Sensor MWD Monitoring for a Roof Bolter

**DOI:** 10.3390/s26196260

**Published:** 2026-10-02

**Authors:** Jianju Ren, Yuxuan Li, Xinpeng Yan, Bao Zhang, Wenlong Zhang

**Affiliations:** 1 School of Resource Engineering, Xi’an University of Architecture and Technology, Xi’an 710055, China; rjj18049300680@xauat.edu.cn (J.R.); liyuxuan@xauat.edu.cn (Y.L.); yxp032666@xauat.edu.cn (X.Y.); zhangbao122320@xauat.edu.cn (B.Z.); 2 State Key Laboratory of Materials Processing and Die & Mould Technology, School of Materials Science and Engineering, Huazhong University of Science and Technology, Wuhan 430074, China; 3 School of Smart City Engineering, Qingdao Huanghai University, Qingdao 266427, China

**Keywords:** measurement-while-drilling (MWD), roof bolter, rock identification, drilling parameters, borehole variability

## Abstract

Built-in multi-sensor measurement-while-drilling (MWD) modules on roof bolters can record torque, thrust, rotational speed, and displacement during drilling. This study examines whether identical rock types produce consistent MWD responses when tested through different boreholes in separate composite specimens. Three laboratory-scale composite specimens containing yellow sandstone (YS), dark-colored red sandstone (DCRS), and light-colored red sandstone (LCRS) were drilled with the same integrated rig, and the sensor responses and specific energy were compared using descriptive distributions, mean values, and relative differences. The results descriptively indicate that torque and thrust respond more clearly to lithological changes than rotational speed, while torque is generally the most stable indicator. Under nominally identical lithological conditions, responses varied across boreholes in separate composite specimens, with reported relative differences reaching approximately 30% in some comparisons. Because one borehole was drilled per specimen, this preliminary study identifies a cross-specimen reproducibility limitation only; it does not estimate within-rock-mass variability or validate a transferable prediction model. The practical implication is limited to showing that single-borehole formulas should not be transferred without calibration, while normalization, hierarchical modeling, and larger multi-borehole validation remain future research directions.

## 1. Introduction

The roof bolter is used in various geotechnical engineering [1] scenarios as part of an essential drilling process before installing a bolt/anchor cable. The main purpose of drilling is to create space for the installation of the bolt/anchor cable and anchoring agents. However, these drill holes prepared for the processes have another additional function, that is, by using the performance of the drill rig or the medium during drilling, to determine the properties [2] and structure [3,4] of the drilled rock, which is called measurement-while-drilling (MWD) [5,6]. The obtained properties and structure of the roof rock can be used to obtain geological data to evaluate the stability of the roof [7] and provide a design basis for support [8]. Since the monitoring work does not affect the completion of the main processes [9], it has received much attention and research from many scholars in recent years [10].

Recent MWD research has advanced the understanding of drilling mechanisms, sensor integration, data transmission, and signal interpretation [11,12,13,14,15,16]. Previous studies have investigated drilling speed, thrust, torque, penetration rate, drilling energy, and data normalization for geological characterization and rock-property estimation [17,18,19,20]. Other studies have further explored vibration- and pressure-based MWD measurements under specific engineering conditions [21,22,23]. Despite these advances, the transferability of indicator–property relationships across boreholes in separate specimens remains insufficiently tested.

A key unresolved issue is whether a monitoring indicator calibrated in one specimen borehole remains reliable in another specimen borehole containing the same rock type. Drilling parameters are affected not only by lithology but also by rig power transmission, bit–rock friction, specimen heterogeneity, operator practice, and other boundary conditions [24,25,26,27,28,29,30]. Consequently, a formula that performs well in one test may not generalize to a borehole in another nominally equivalent specimen. This preliminary descriptive study therefore focuses on cross-specimen borehole response inconsistency and on the limits of transferring MWD indicators beyond the conditions in which they were calibrated.

The research problem is the limited evidence for reproducibility of MWD signals for the same rock type across boreholes in separate specimens. We descriptively compare responses from three composite specimens and explicitly distinguish between (i) lithology discrimination within one specimen and (ii) the transferability of an indicator between specimen boreholes. The study does not develop or validate a universal prediction formula, a normalization procedure, or a hierarchical model; it identifies an experimental boundary condition that must be addressed before such methods are generalized.

## 2. Materials and Methods

**Figure 1:**
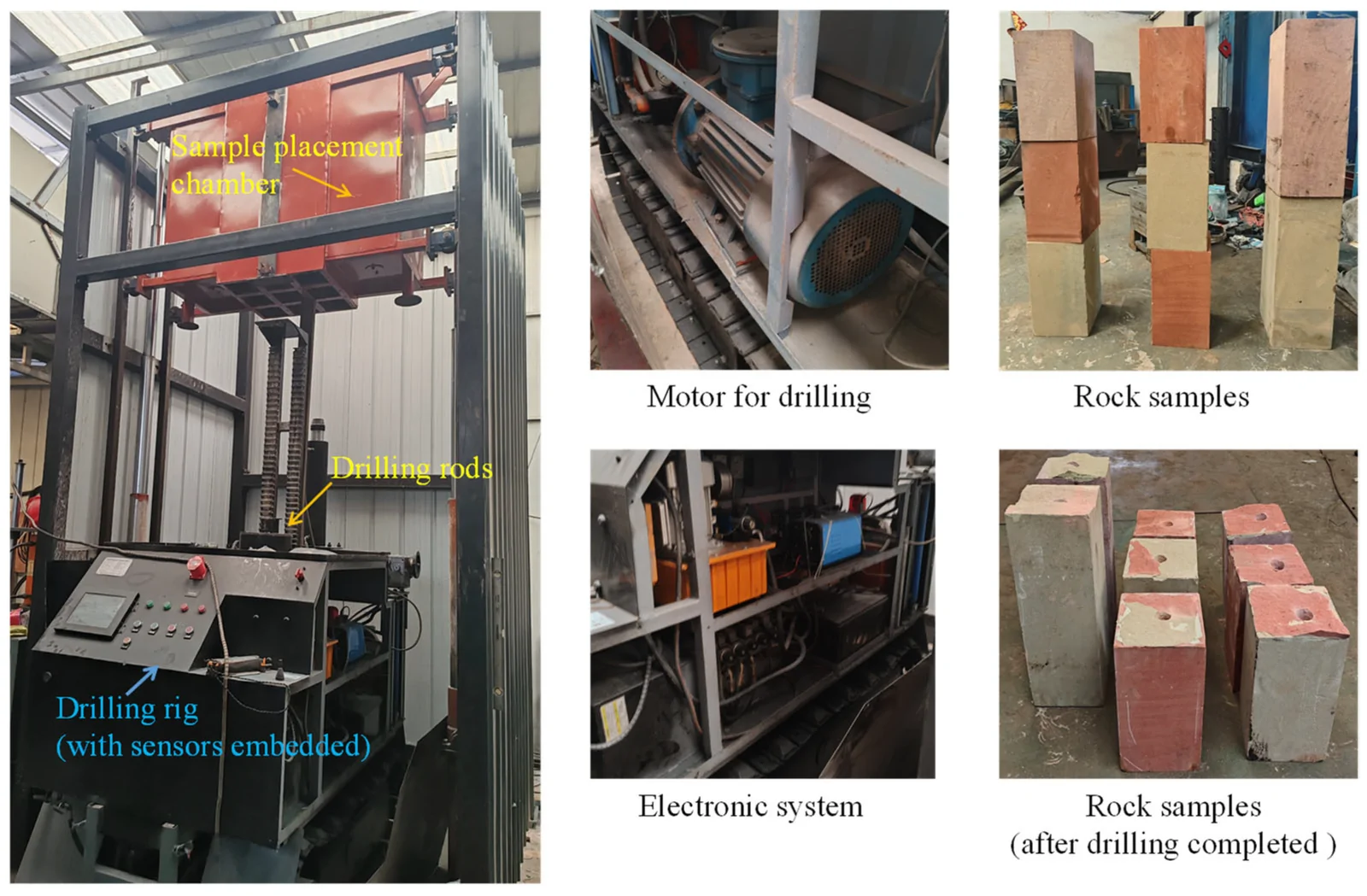
Experimental system.

In order to obtain the application of MWD technology in a roof bolter, especially to determine whether the performance of the same rock is consistent in different boreholes, the experimental device and samples shown in Figure 1 were adopted. The drill rig used in the experiment was an integrated one, which is more stable and reliable than the single roof bolter in previous studies. Moreover, sensors such as the torque sensor, thrust sensor, rotational speed sensor and displacement sensor were built into the drill rig. The upper outlet of the drill rig is where the drill rods are installed and connected. The power provided by the drill rig is transmitted to the drill bit through the drill rods, thereby drilling into the rock. The top of the device is the sample placement chamber, which can store multiple samples at one time. The samples are generally square in shape to facilitate better fixation when rotated, in order to prevent the samples from rotating along with the drill bit and the drill rods.

The power supply voltage of the experimental drill rig is 380 V/660 V, with a power of 11 kW. The rated rotational speed is 320 r/min, and the rated torque is 140 N·m. The sampling frequency of the signals is 10 Hz, and the signals of each sensor are transmitted in through a wired transmission method. The displacement sensor is a rope-pulling type, and the current drilled depth is obtained by measuring the extended distance of the rope during drilling. The rotational speed sensor is of Hall type, with an effective measuring range of up to 2000 r/min, which can fully meet the requirements of the experiment. The thrust and torque sensor is an integrated installation type, which obtains feedback by measuring the thrust and torque of the shaft. The effective measuring range of the thrust sensor can reach 1000 kN, and the effective measuring range of the torque sensor can reach 500 N·m. The performance of the roof bolter when drilling different rocks was fed back through the data of these four sensors for subsequent analysis and use.

A total of three composite specimens were prepared, consisting of three rock types: yellow sandstone (YS), dark-colored red sandstone (DCRS), and light-colored red sandstone (LCRS). The first specimen contained YS in the shallow part and LCRS in the deep part (50 cm each; total height 100 cm). The second contained DCRS, YS, and DCRS in the shallow, middle, and deep parts, respectively (33 cm each; total height 100 cm). The third contained YS, DCRS, and LCRS in the shallow, middle, and deep parts, respectively (33 cm each; total height 100 cm). The uniaxial compressive strengths of YS, DCRS, and LCRS were 20, 35, and 65 MPa, respectively. One designated borehole was drilled through each composite specimen. Thus, the cross-specimen borehole comparisons in this paper refer to boreholes located in different composite specimens, not repeated boreholes in a single intact rock mass.

The same integrated rig, drill-bit/drill-rod configuration, sampling frequency (10 Hz), and nominal operating procedure were used for all tests. Rotational speed, thrust, torque, and displacement were recorded rather than independently fixed; the penetration rate was calculated from displacement. The operator and environmental conditions were not independently randomized or logged, and temperature and humidity were not measured. These uncontrolled factors are treated as possible sources of cross-specimen variability and are included as limitations of the present laboratory study.

Specific energy (SE) was calculated according to Teale [25] as

$$\mathrm{SE}=\frac{F}{A}+\frac{2\pi \mathrm{nT}}{\mathrm{Av}},$$

where *F* is the thrust, *T* is the torque, *n* is the rotational speed expressed in revolutions per unit time, *v* is the penetration rate, and *A* is the borehole cross-sectional area determined from the drill-bit diameter.

To quantify the difference between any two reported mean values, the relative difference was calculated as

$$\mathrm{Relative}\mathrm{difference}=\frac{\left|x-x_{\mathrm{ref}}\right|}{x_{\mathrm{ref}}}\times 100\%,$$

where *x*_ref_ denotes the mean value of the first-listed condition or specimen. The reference used for each comparison is specified in the accompanying text or figure.

Sample 2 contained two DCRS intervals. For cross-specimen comparisons of the DCRS intervals, the superficial DCRS interval was selected because it was the first complete DCRS interval encountered along the drilling direction. By contrast, the deep DCRS interval was retained for the within-specimen analysis presented in the subsequent figures, but it was not treated as a second independent specimen replicate.

## 3. Results and Discussion

**Figure 2:**
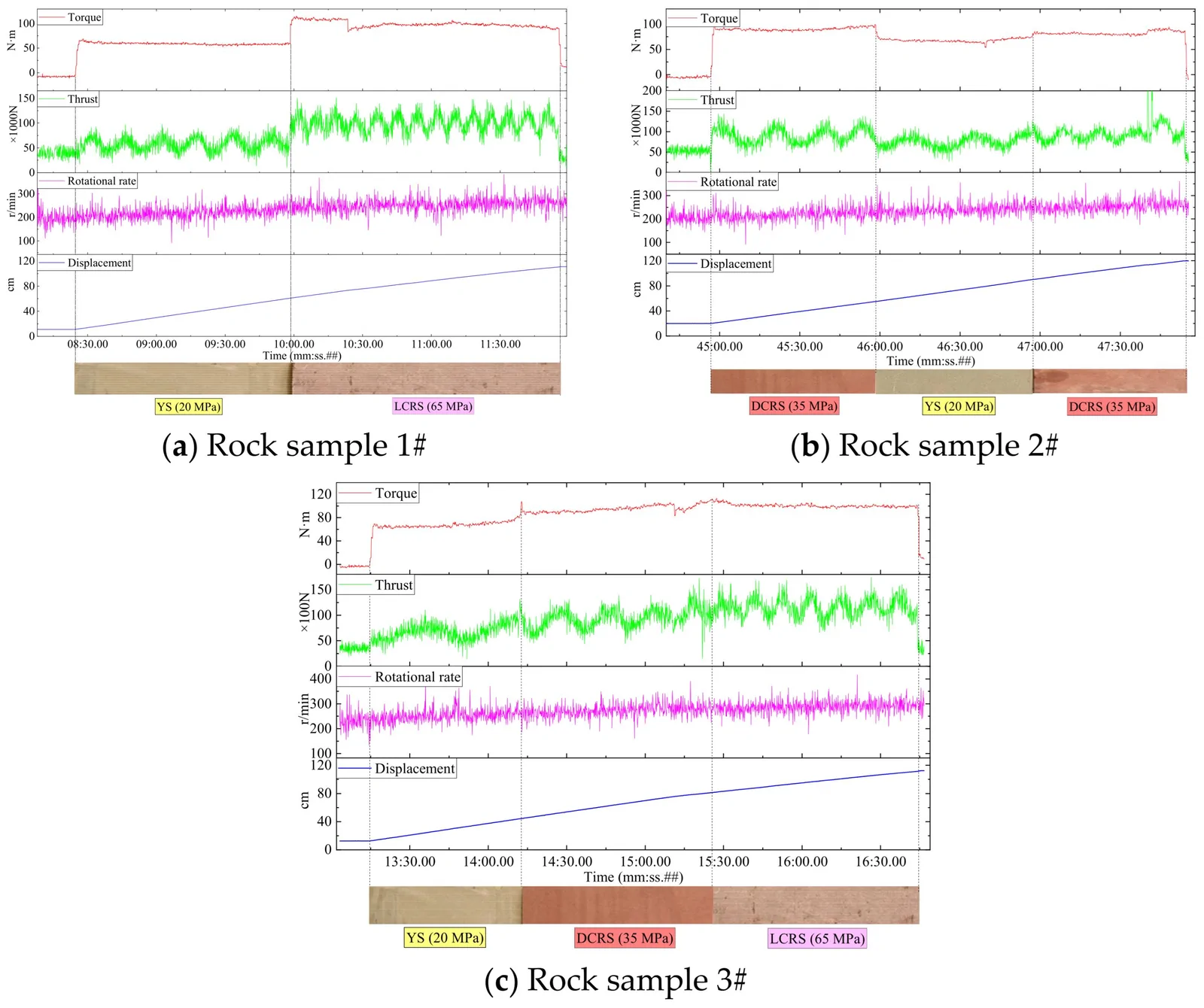
Monitoring results of different indicators for each sample.

After careful and strict drilling and synchronous signal acquisition, the monitoring-while-drilling results of three samples were obtained, as shown in Figure 2, from the results of sample 1#. During the whole drilling process, the rotational speed was basically the same at 200 r/min, and it increased slightly during deep drilling. The thrust fluctuated back and forth, but it still reacted where the rock changes. LCRS with greater strength has a larger thrust value. The torque was relatively stable, and the difference seemed to be larger, but in the drilling process of LCRS, it was divided into two sections; one was larger and the other was smaller, and the discrimination was good overall. The discrimination degree of sample 2# was smaller than that of sample 1#. Whether it was thrust or torque, the difference was not very large. Of course, this was consistent with the small difference between the compressive strength of the two kinds of rocks in sample 2#. The difference in indicators of sample 3# was not as large as it seems, but the results were consistent with the actual sample situation. As the compressive strength continued to increase, the thrust and torque of the three types of rocks also increased in stages. In general, the depth monitoring results of the three samples were in line with the actual situation, and there was no obvious change in the rotational speed, but the thrust and torque had obvious different values when drilling different rocks. The stability of thrust index was poor, and the stability and difference in torque index were the best from the visual observation. The displacement of drilling per unit time (propelling speed) is not shown in Figure 2, and it was impossible to determine the propelling speed of different rocks, which will be shown and compared in the subsequent results.

**Figure 3:**
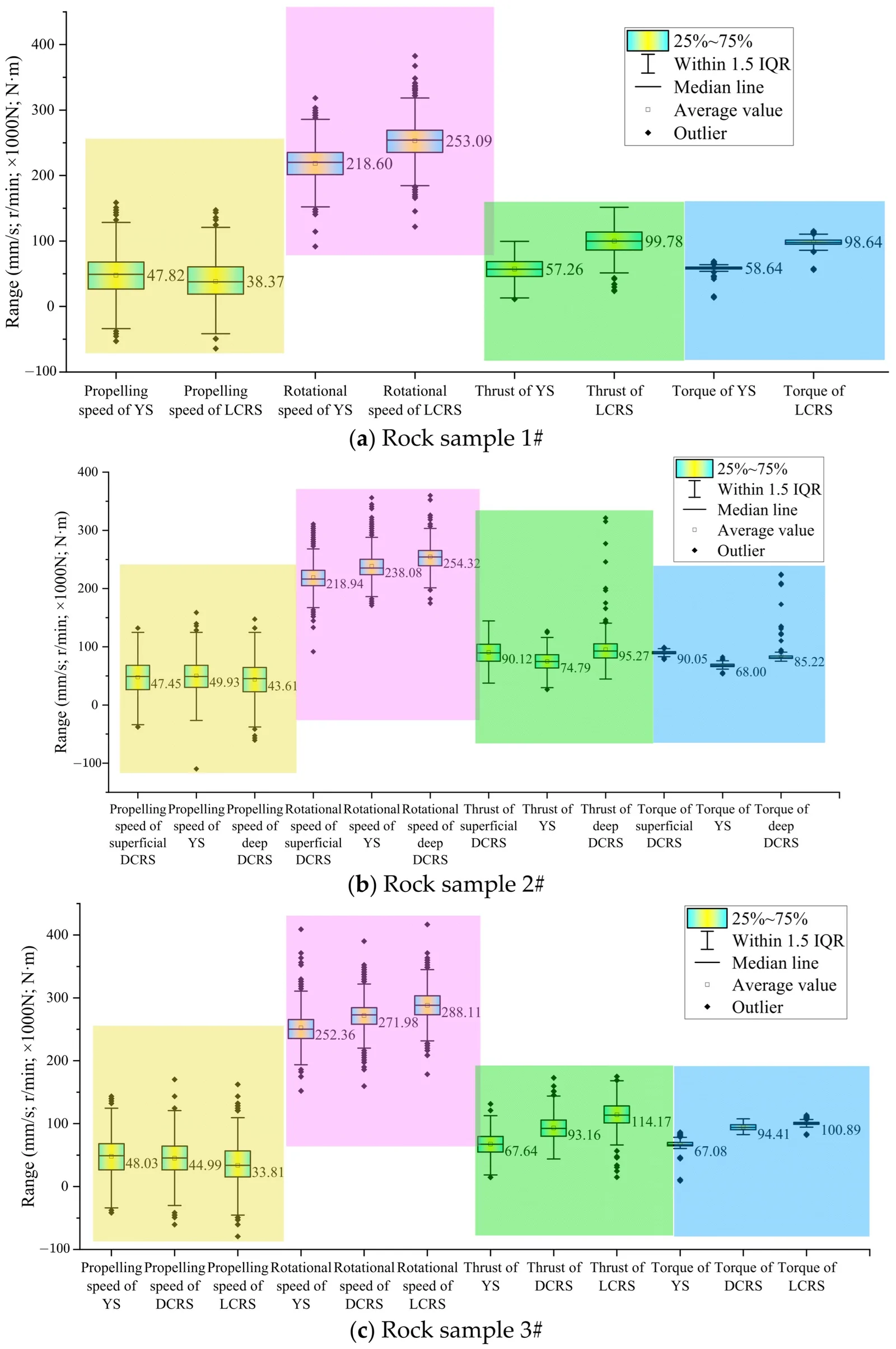
The indicator comparison results of different rocks in the same sample.

Indicator comparison results of different rocks in same composite specimen are denoted in Figure 3. The figure mainly shows the differences in the response of different rocks in the same composite specimen to the indicators of propelling speed, rotational speed, torque and thrust. Figure 3a presents boxplots comparing different drilling indicators for rock sample 1#. The parameters were categorized into four main types. The propelling speed indicators were concentrated in a low-value range with small dispersion, compact box distributions, few outliers, and limited overall fluctuation. The rotational speed indicators were concentrated in a medium-to-high range, exhibiting a much wider distribution range, higher overall box positions, and numerous outliers, indicating strong dispersion. The thrust indicators fell in the medium-to-low range, with relatively concentrated distributions, moderate box widths, and few outliers. The torque indicators were also concentrated in the medium-to-low range, showing low overall dispersion, compact box distributions, and good data stability. Overall, the rotational speed indicators were numerically higher than the other indicators, while the propelling speed indicators showed the lowest overall level. The distribution characteristics and fluctuation differences of different drilling indicators were clearly illustrated.

Figure 3b displays boxplots comparing different drilling indicators for rock sample 2#, categorized by drilling conditions and indicator types. The propelling speed indicators were in a low-value range with concentrated distributions, small box widths, a few outliers, and limited overall fluctuation. The rotational speed indicators were concentrated in a medium-to-high range, with a wide distribution range, high box positions, numerous outliers, and pronounced dispersion differences across different conditions. The thrust indicators fell in the medium-to-low range, with large distribution differences across conditions; some indicators had wider boxes, increased dispersion, and more outliers. The torque indicators were concentrated in the medium-to-low range, with high dispersion under certain conditions, larger box widths, and more outliers than in other conditions. Overall, rotational speed indicators remained the highest, while thrust and torque indicators showed more pronounced distribution differences across different conditions.

Figure 3c shows boxplots comparing different drilling indicators for rock sample 3#, categorized by indicator type and drilling conditions. The propelling speed indicators were in a low-value range with concentrated distributions, small box widths, a few outliers, and good overall stability. The rotational speed indicators were concentrated in a medium-to-high range, with a wide distribution range, high box positions, numerous outliers, and strong dispersion across different conditions. The thrust indicators fell in the medium-to-low range, with pronounced distribution differences across conditions; some indicators had wider boxes, numerous outliers, and large fluctuation ranges. The torque indicators were concentrated in the medium-to-low range, with relatively concentrated distributions, moderate box widths, few outliers, and good overall stability. Overall, rotational speed indicators were still numerically higher than the other indicators, with distribution differences across drilling conditions being more pronounced in thrust and rotational speed indicators.

**Figure 4:**
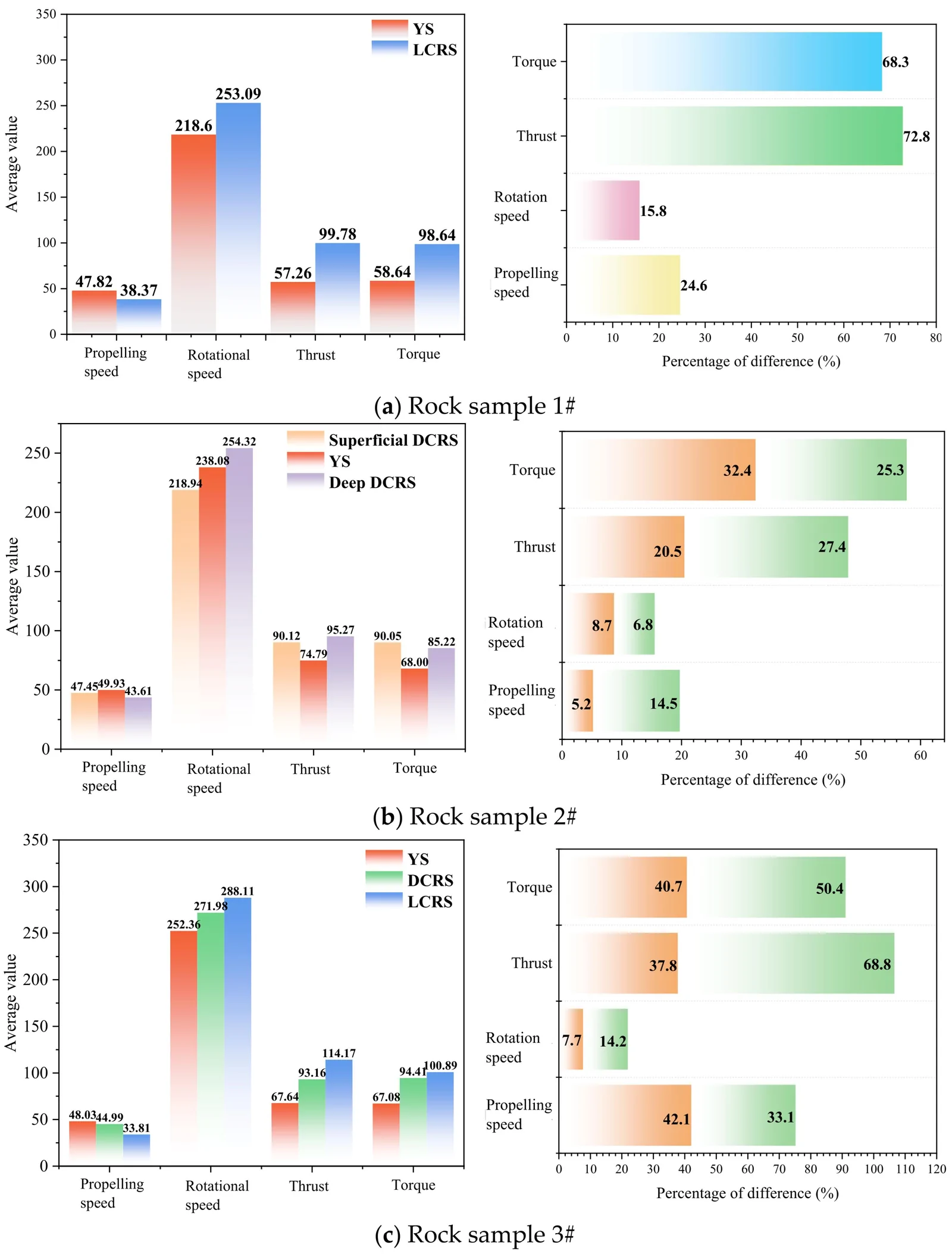
The quantitative comparison results of the indicators for different rocks in the same sample.

The quantitative comparison results of the indicators for different rocks in the same composite specimen are shown in Figure 4. Figure 4a presents the quantitative comparison of various drilling indicators for the No. 1 rock sample, including the statistical analysis of mean values and difference proportions. In terms of the mean values, the penetration rate remained at an overall low level, with values of 47.82 for the YS condition and 38.37 for the LCRS condition, showing a minor discrepancy between the two working conditions. The rotational speed occupied the highest level among all indicators, with averages of 218.6 under the YS condition and 253.09 under the LCRS condition, which were obviously higher than other parameters. For the thrust, the corresponding values were 57.26 (YS) and 99.78 (LCRS), while the torque values were 58.64 (YS) and 98.64 (LCRS). The descriptive proportion analysis showed that thrust had the largest difference proportion (72.8%), followed by torque (68.3%). The difference proportion of the penetration rate was 24.6%, and the rotational speed exhibited the lowest proportion of only 15.8%. On the whole, the thrust and torque under the LCRS condition were higher than those under the YS condition. Although the absolute values of rotational speed differed greatly, its relative proportional difference was small, demonstrating that working conditions imposed a more prominent influence on thrust and torque.

Figure 4b illustrates the quantitative differences in drilling indicators for the No. 2 rock sample under three scenarios, namely the surface DCRS, YS, and deep DCRS conditions. The penetration rates were relatively consistent across groups, with mean values of 47.45 for surface DCRS, 49.93 for YS, and 43.61 for deep DCRS. The rotational speed maintained a high range in all cases, reaching 218.94 (surface DCRS), 238.08 (YS), and 254.32 (deep DCRS). The thrust values were 90.12, 74.79 and 95.27 for the three conditions, respectively, and the corresponding torque values were 90.05, 68.00 and 85.22. According to the difference proportion statistics, the torque showed difference proportions of 32.4% and 25.3%, and the thrust had proportions of 20.5% and 27.4%. The penetration rate yielded low difference proportions of 5.2% and 14.5%, and the rotational speed had the smallest variation at 8.7% and 6.8%. The rotational speed under the deep DCRS condition was the highest, whereas the thrust and torque under the YS condition were relatively low. The variations in indicators induced by different working conditions were more remarkable in thrust and torque parameters.

Figure 4c summarized the quantitative comparison results of drilling indicators for the No. 3 rock sample among the YS, DCRS and LCRS working conditions. The penetration rates were generally low and similar, with averages of 48.03 (YS), 44.99 (DCRS), and 33.81 (LCRS). The rotational speed was always dominant with high magnitudes of 252.36, 271.98 and 288.11 in sequence. The thrust increased obviously from 67.64 (YS) to 93.16 (DCRS) and 114.17 (LCRS), and the torque also rose from 67.08 to 94.41 and 100.89 correspondingly. The difference proportion analysis revealed that the thrust had the largest variation proportions of 37.8% and 68.8%, and the torque presented proportions of 40.7% and 50.4%. The penetration rate had moderate difference proportions of 42.1% and 33.8%, and the rotational speed possessed relatively low values of 7.7% and 14.2%. All drilling parameters reached their peak values under the LCRS condition and remained the lowest under the YS condition. Working conditions exerted the largest descriptive difference on the thrust, with far higher difference proportions than other indicators.

Based on the comprehensive analytical results of the three rock samples in Figure 4, for each rock type, the responses of drilling indicators were inconsistent under the same specimen environments and working conditions. The rotational speed possessed the overall maximum absolute values but relatively low proportional variations, reflecting its favorable stability against condition changes. By contrast, the thrust and torque showed dramatic fluctuations in absolute values and difference proportions, indicating their high sensitivity to various working scenarios. The penetration rate stayed at a low level with medium difference proportions without extreme fluctuations. These findings suggested that the mechanical drilling responses of the rock were not merely determined by lithology. Specimen characteristics and operational conditions also exerted considerable impacts on key drilling parameters, which should be comprehensively considered in the optimization of engineering drilling schemes.

Previous MWD studies used drilling variables to detect interfaces, characterize geology, or predict rock properties under defined rigs and site-specific calibration conditions [17,18,19,20,24,29,30,31,32]; filtering and normalization have also been used to reduce systematic drilling effects [18]. Their accuracy or classification metrics are not directly comparable with the relative-difference measure used here. In this preliminary dataset, same-lithology cross-specimen differences reached 30.6% for thrust under YS, 24.2% for rotational speed under DCRS, and 16.0% for torque under YS. Torque generally showed smaller cross-specimen differences than thrust or rotational speed. Thus, the present study does not claim superior prediction performance; it adds a descriptive reproducibility constraint to earlier calibrated MWD applications and supports normalization and independent multi-borehole validation before transfer.

**Figure 5:**
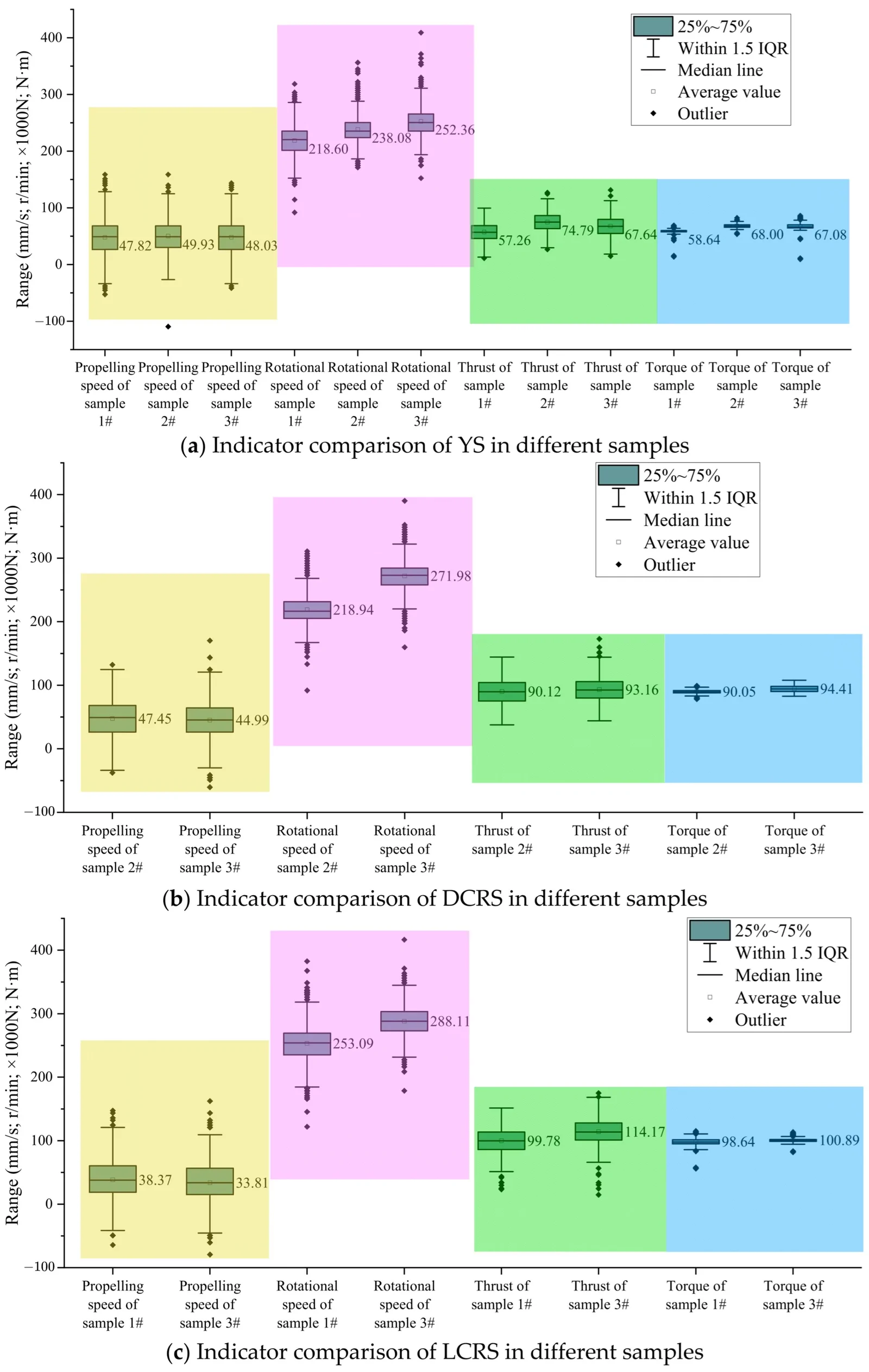
The indicator comparison results of the same rock across different composite specimens.

The indicator comparison results of the same rock across different composite specimens are calculated and displayed in Figure 5. Figure 5a presents boxplots comparing the drilling indicators of different rock samples under the YS condition, categorized into four types: propelling speed, rotational speed, thrust, and torque. The propelling speed indicators are concentrated in a low-value range with small dispersion, compact box distributions, and only a few outliers, showing limited fluctuation and small descriptive differences among rock samples. The rotational speed indicators are concentrated in a medium-to-high range, with higher overall box positions, wider distribution ranges, and numerous outliers, and differences in box positions and dispersion existing among different composite specimens. The thrust indicators fall in the medium-to-low range with moderate box widths and relatively concentrated distributions, showing minor differences among samples. The torque indicators are also concentrated in the medium-to-low range with low dispersion and compact box distributions, presenting good stability and small descriptive differences among samples. Overall, the rotational speed indicators are numerically higher than the other indicators, while the propelling speed indicators show the lowest level, and the differences among the rock samples are more pronounced in rotational speed.

Figure 5b displays boxplots comparing the drilling indicators of different rock samples under the DCRS condition. The propelling speed indicators are in a low-value range with concentrated distributions, small box widths, and a few outliers, showing limited fluctuation and minor differences among samples. The rotational speed indicators are concentrated in a medium-to-high range, with high box positions, wide distribution ranges, numerous outliers, and obvious differences in dispersion among samples. The thrust indicators fall in the medium-to-low range with moderate box widths and relatively concentrated distributions, showing certain differences in thrust levels among samples. The torque indicators are concentrated in the medium-to-low range with low dispersion and compact box distributions, presenting good stability and small descriptive differences among samples. Overall, the rotational speed indicators remain the highest, and the differences among samples are more prominent in rotational speed and thrust indicators.

Figure 5c shows boxplots comparing the drilling indicators of different rock samples under the LCRS condition. The propelling speed indicators are in a low-value range with concentrated distributions, small box widths, and a few outliers, showing limited fluctuation and minor differences among samples. The rotational speed indicators are concentrated in a medium-to-high range, with high box positions, wide distribution ranges, numerous outliers, and certain differences in dispersion among samples. The thrust indicators fall in the medium-to-low range with moderate box widths and relatively concentrated distributions, showing obvious differences in thrust levels among samples. The torque indicators are concentrated in the medium-to-low range with low dispersion and compact box distributions, presenting good stability and small descriptive differences among samples. Overall, the rotational speed indicators are numerically higher than the other indicators, and the differences among rock samples are more pronounced in thrust indicators.

**Figure 6:**
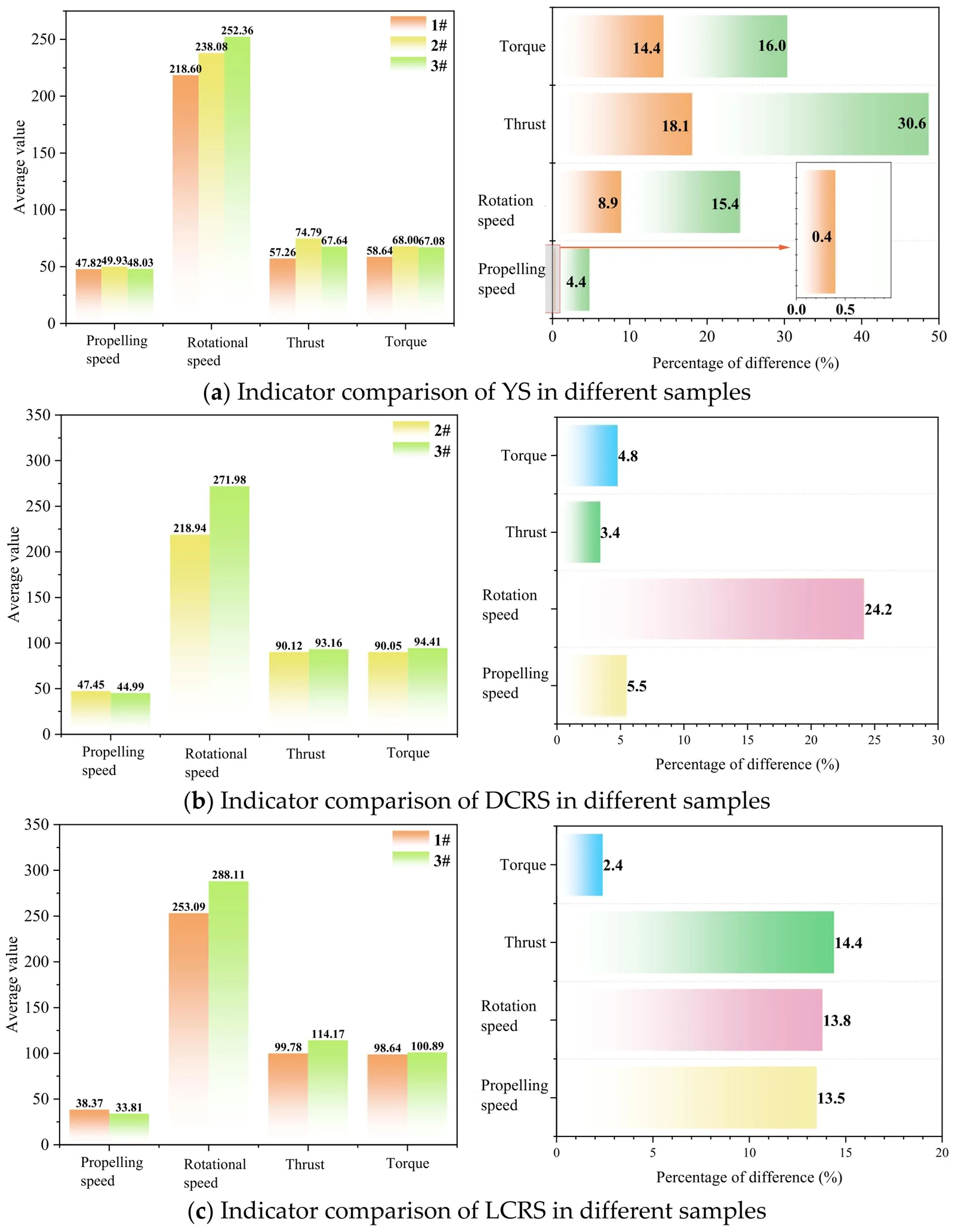
A quantitative comparison of drilling indicators for the same rock across different composite specimens.

Figure 6a presents the descriptive comparison of drilling indicators for samples 1–3 under the YS condition. The mean propelling speeds were 47.82, 49.93, and 48.03; rotational speeds were 218.60, 238.08, and 252.36; thrust values were 57.26, 74.79, and 67.64; and torque values were 58.64, 68.00, and 67.08 for samples 1, 2, and 3, respectively. Using sample 1 as the reference, the difference proportions for samples 2 and 3 were 4.4% and 0.4% for propelling speed, 8.9% and 15.4% for rotational speed, 30.6% and 18.1% for thrust, and 16.0% and 14.4% for torque. Thrust therefore showed the largest cross-specimen difference in this comparison, whereas propelling speed showed the smallest differences.

Figure 6b shows the quantitative differences in drilling indicators between samples 2# and 3# under the DCRS condition. The propelling speed values were 47.45 for sample 2# and 44.99 for sample 3#, with a minor discrepancy between the two. The rotational speed remained at a high level, with averages of 218.94 (2#) and 271.98 (3#), presenting a notable difference. The thrust values were 90.12 (2#) and 93.16 (3#), which were close to each other, and the torque values were 90.05 (2#) and 94.41 (3#), also showing a small difference. According to the difference proportion analysis, the rotational speed had the highest proportion at 24.2%, followed by propelling speed (5.5%), torque (4.8%), and thrust (3.4%). Overall, the rotational speed showed the largest descriptive difference by sample differences, while the thrust and torque exhibited relatively stable performance with low difference proportions.

Figure 6c illustrated the quantitative comparison of drilling indicators between samples 1# and 3# under the LCRS condition. The propelling speed values were 38.37 for sample 1# and 33.81 for sample 3#, with a small difference. The rotational speed was at a high level, with averages of 253.09 (1#) and 288.11 (3#), showing a noticeable discrepancy. The thrust values were 99.78 (1#) and 114.17 (3#), presenting a considerable difference. The torque values were 98.64 (1#) and 100.89 (3#), which were highly consistent. The difference proportion analysis indicated that the thrust had the highest proportion at 14.4%, followed by rotational speed (13.8%) and propelling speed (13.5%), while the torque had the lowest proportion of only 2.4%. In general, the torque showed excellent stability between the two samples, whereas the thrust, rotational speed, and propelling speed showed larger descriptive differences by sample variations, with difference proportions all exceeding 13%.

The comprehensive quantitative results in Figure 6 revealed that even under the same working condition, the drilling indicators of different rock samples exhibited obvious inconsistencies. The mean indicator values varied among different composite specimens under the same condition, and the sensitivity of each indicator to sample differences also differed, with rotational speed and thrust generally showing higher difference proportions, while torque demonstrated better stability in most conditions. This indicated that even for the same type of rock, the mechanical responses were not entirely consistent, and the heterogeneity among samples directly affected the key parameters during drilling. Therefore, in engineering applications and experimental analyses, the fluctuation of indicators caused by sample differences must be fully considered to avoid the one-sidedness of a single test result.

**Figure 7:**
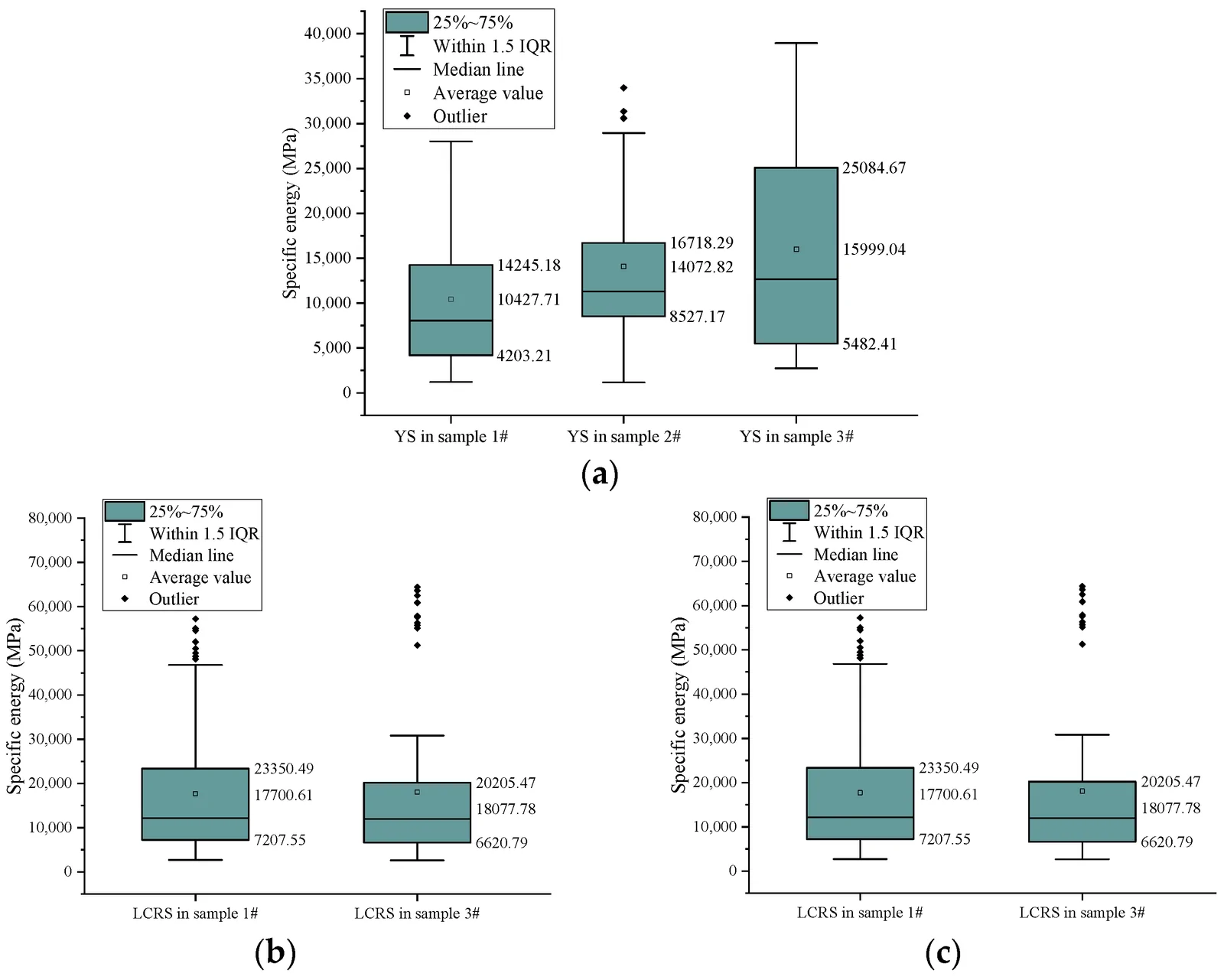
The specific energy comparison results of the same rock across different composite specimens. (**a**) The specific energy comparison of YS in different samples; (**b**) the specific energy comparison of DCRS in different samples; (**c**) the specific energy comparison of LCRS in different samples.

Figure 7 presents descriptive specific-energy distributions for the same lithology across boreholes in separate composite specimens. Under the YS condition, all three specimens showed broad distributions and upper outliers; sample 3 had the highest mean and median and the widest spread. Under DCRS, samples 2 and 3 also showed broad distributions, with higher central values and greater dispersion in sample 3. Under LCRS, the comparison is between samples 1 and 3, consistent with the specimen composition described in Section 2; sample 1 had a slightly higher center, while both specimens showed wide dispersion and upper outliers. These patterns are descriptive cross-specimen differences and do not constitute inferential evidence of within-rock-mass borehole variability.

Taken together, the results should be interpreted as preliminary cross-specimen descriptive evidence. Rotational speed differed across same-lithology specimen boreholes and was not independently fixed, so it may contribute to the observed variability. Earlier MWD studies demonstrated interface detection, geological characterization, or property prediction within defined calibration settings [18,24,29,30,31,32]. Here, relative differences of up to approximately 30% show that a fixed formula calibrated in one borehole cannot be assumed to transfer to another specimen borehole. The current dataset cannot determine causation, quantify repeated-borehole variability within one intact rock mass, or validate normalization or hierarchical models. Those questions require controlled repeated boreholes, independently prepared specimens, logged operating conditions, and held-out validation.

## 4. Study Limitations and Future Work

Because only one borehole was drilled in each composite specimen (three specimens in total), the depth-wise sensor readings are repeated observations rather than independent specimen-level replicates. We therefore report descriptive distributions, means, and relative differences, but do not claim *p*-values, confidence intervals, ANOVA results, effect sizes, or statistical significance from this dataset. The present work is a preliminary descriptive study and does not demonstrate a normalization procedure or hierarchical model with the available data. Future work requires multiple boreholes per rock type and multiple independently prepared specimens for inferential testing and power analysis. A future analysis could normalize each borehole by a prespecified within-test reference segment and fit a hierarchical model with rock type as a fixed effect and specimen, borehole, and operator as random effects, followed by validation on held-out boreholes.

## 5. Conclusions

This preliminary laboratory study descriptively compared three composite specimens containing three rock types and one designated borehole per specimen. Torque and thrust provided clearer lithological responses than rotational speed, and torque generally showed the most concentrated distributions. Nevertheless, the same rock type produced different responses across specimen boreholes, with relative differences of up to approximately 30% in the reported comparisons. The finding is limited to a cross-specimen reproducibility constraint; it is not a universal estimate of within-rock-mass variability and does not validate a transferable prediction method. The practical implication is limited to cautioning against transferring single-indicator or fixed-formula predictions between boreholes without calibration. Repeated boreholes, logged operating and environmental conditions, inferential analysis, and independent validation are required before the indicators can be generalized.

## Data Availability

The data presented in this study are available from the corresponding author upon reasonable request. The data are not publicly available due to ongoing research and data management considerations.

## Abbreviations

The following abbreviations are used in this manuscript:

YS	Yellow sandstone
DCRS	Dark-colored red sandstone
LCRS	Light-colored red sandstone
